# Variations in Bacterial and Archaeal Communities and Baijiu Metabolites by Distillery and Pit Age

**DOI:** 10.3390/foods15152657

**Published:** 2026-07-28

**Authors:** Yanping Xu, Yue Deng, Wenqi Xiao, Xudong Zhou, Xianli Guo, Jianxiang Yi, Linting Zhang, Xian Peng, Jingcheng Liu, Qiang Li

**Affiliations:** 1 School of Food and Biological Engineering, Chengdu University, Chengdu 610106, China; xuyp0422@163.com (Y.X.); xwq990713@126.com (W.X.); 18782136172@163.com (X.G.); jxy18322946488@163.com (J.Y.); 15700602958@163.com (L.Z.); pq@163.com (X.P.); 2 School of China Alcoholic Drinks, Luzhou Vocational and Technology College, Luzhou 646000, China; scudeng@hotmail.com (Y.D.); zxd7476@163.com (X.Z.)

**Keywords:** bacterial community diversity, pit age, differential metabolites, pit mud, baijiu, archaeal community diversity

## Abstract

Pit mud (PM) harbors abundant bacteria and archaea, shaping Baijiu’s flavor and adapting to high-ethanol and acidic conditions. This study integrated 16S rRNA sequencing, physicochemical analysis, and non-targeted metabolomics. The results showed that the 30-year-old Zhuyechun PM had higher levels of humic acid, while the 20-year-old Fengchun and Bitanchun PM exhibited near-neutral pH, which is favorable for Clostridia and methanogens. Canonical correspondence analysis revealed *Rummeliibacillus* and *Caproiciproducen* positively correlated with organic carbon, humic acid, and available phosphorus, whereas *Methanosarcina* showed negative correlations. In Baijiu, 1206 non-volatile signals were identified, with unsaturated fatty acid biosynthesis as a key flavor pathway. Methanogens significantly associated with key metabolites (*p* < 0.05). These findings reveal the correlation between the PM microecosystem and Baijiu quality, providing insights for the development of high-quality Baijiu.

## 1. Introduction

Baijiu is one of the six major distilled spirits in the world, and its solid-state fermentation process is closely related to China’s long history and culture [1,2]. Strong-flavor Baijiu (SFB) is highly favored by consumers for its distinctive style and rich aroma, accounting for 70% of the Chinese Baijiu market [3,4]. SFB is one of the four fundamental aroma types. Influenced by the traditional Baijiu brewing process, the fermented grains are typically a mixture of sorghum, wheat, rice, glutinous rice, and corn. The complex microbial communities across the entire fermentation system jointly drive saccharification, fermentation, and esterification, and the distinctive microbes in PM further endow the finished liquor with unique flavor characteristics [5,6,7].

PM, as the core site for SFB, serves not only as a habitat for microorganisms but also as a complex microbial system [8]. The functional microbial communities formed in PM through natural selection and interactions exhibit diversity and structural establishment influenced by physicochemical factors of the PM, such as humic acid and pH [9]. Additionally, the age of the fermentation pit, surrounding geographical environment, and differences in metabolic products significantly affect the quality of Baijiu [10,11]. Microbial communities in pits of different ages show distinct differentiation, often displaying alliances among microorganisms that collaborate to regulate environmental changes [12].

With the widespread application of metabolomics technology in the field of Baijiu, the non-volatile metabolites in the liquor are receiving increasing attention. Typical non-volatile components such as organic acids, phenolics, and lipids not only participate in the synthesis and transformation of flavor substances, but also significantly affect the sensory characteristics of Baijiu, such as taste and fullness, playing a key role in the formation of the overall style of the liquor [13,14]. Non-targeted metabolomics is a scientific field focused on the detection and analysis of metabolites [15]. In recent years, in the study of Baijiu, chemical isotope labeling combined with liquid chromatography-mass spectrometry (LC-MS) has identified hundreds of non-volatile compounds in different aroma types of Baijiu. Non-targeted metabolomics technology has also been used for grading Baijiu and distinguishing authenticity [16]. Additionally, non-targeted metabolomics has been widely applied to study the dynamic metabolic processes during Baijiu fermentation [17,18]. This experiment conducted a non-targeted metabolomics analysis on liquor samples, aiming to qualitatively characterize the non-volatile substances in the liquor and screen for differential metabolites related to Baijiu.

Mianzhu City in Sichuan Province is renowned as the hometown of fine liquor, boasting abundant resources in SFB. The Baijiu industry has driven the local economy, with different qualities of SFB originating from the use of long-term cycled old and new fermentation pits [19]. Traditional solid-state fermentation often results in inconsistent batch-to-batch product quality, while the aging and degradation of tank sludge can also lead to a decline in wine quality and inhibit the growth of beneficial microorganisms; therefore, a solution is urgently needed. Given the importance of microorganisms in the fermentation process, the use of functional microorganisms for artificial fermentation regulation has received significant attention. Previous studies have conducted preliminary analyses on the physicochemical properties, microbial community structures, and metabolic characteristics of PM across different PM [20,21,22,23]. Other investigations have examined the effects of PM properties on the spatial heterogeneity of bacterial communities and volatile metabolites [24]. However, studies that simultaneously investigate the potential contribution of non-volatile metabolites in Baijiu fermented using the same PM remain scarce. Critically, as the PM age increases, how the SFB microecosystem evolves and its correlation with non-volatile metabolites remain largely unexplored.

This study aimed to: (1) Comparatively analyze the compositional and diversity differences in bacterial and archaeal communities in PM from different distilleries; (2) Investigate the reassembly and succession of bacterial and archaeal communities in PM across different PM, alongside the concomitant changes in volatile metabolites of SFB; (3) Elucidate the intercorrelation mechanisms among physicochemical factors, bacterial and archaeal community composition, and volatile metabolites of strong-flavor Baijiu across different distilleries and PM. Multi-dimensional integrative analyses were conducted to investigate the diversity of bacteria and archaea across different environments and pit ages, as well as the associated changes in non-volatile metabolites of Baijiu. Furthermore, a three-dimensional correlation network of “environmental factors–microorganisms–metabolites” was constructed by incorporating physicochemical indicators. These investigations will contribute to a better understanding of the PM microbiome structure and the ecological rules governing community assembly, thereby holding significant implications for pit management and sustainable brewing.

## 2. Materials and Methods

### 2.1. Study Site and Sample Collection

The samples in this study were collected from Mianzhu City, Sichuan Province, China, which is located in a humid subtropical monsoon climate zone (103°54′–104°20′ E, 31°09′–31°42′ N). The sampling sites were selected from Sichuan Mianzhu Bitanchun Liquor Co., Ltd., Sichuan Mianzhu Zhuyechun Liquor Co., Ltd., and Mianzhu Fengchun Liquor Co., Ltd., (All liquor samples analyzed in this study were sourced from Mianzhu City, Sichuan Province, China.) all of which are situated within the surrounding area of the Mianzhu high-quality Baijiu industrial cluster. They are adjacent to the renowned Jiannanchun liquor distillery, with abundant water resources and rich mineral content. The Baijiu samples were produced from the same fermentation pit and the same fermentation batch. The raw material formula was uniformly composed of a five-grain blend of sorghum, wheat, rice, glutinous rice, and corn. The selected fermentation pits adopted the traditional solid-state continuous fermentation process, with a fermentation period of 75 days, and the distillation operations were performed identically. Upon completion of fermentation, the middle-section distillate was collected into sealed bottles and stored away from light during transportation. Meanwhile, PM samples were simultaneously collected from the fermentation pit workshops of the three distilleries. Four independent production pits were selected: 20-year-old pits were sampled from Bitanchun and Fengchun distilleries, while 20-year-old and 30-year-old pits were sampled from Zhuyechun Distillery. Sampling was performed using a stratified random sampling method [25,26]. Sampling points were established according to the previously described procedure. Sampling points were set on the pit wall at three depths: the upper layer at 12 cm below the pit surface, the middle layer at 1.5 m below the pit surface, and the lower layer at 2–3 cm above the pit bottom. Three random samples were collected from each layer, and samples from different layers were mixed homogeneously to obtain 100 g of PM sample. The sample was then placed in sterile bags, transported to the laboratory with ice packs, and stored at −20 °C. Under identical experimental conditions, the entire procedure was repeated three times as independent samplings (*n* = 3).

### 2.2. Determination of Physicochemical Properties of PM

Measuring the available phosphorus in PM using previous methods [27] led to the determination of the pH of PM using the potentiometric method [28]. The organic carbon content of PM was determined using the potassium dichromate oxidation-external heating method [29]. The humic acid content of PM was measured by potassium dichromate oxidation-ammonium ferrous sulfate standard solution titration [30]. The total phosphorus content of PM was determined using NaOH fusion and measured at a wavelength of 700 nm by the molybdenum-antimony anti-spectrophotometric method [31]. The total potassium content of PM was measured by the NaOH alkaline fusion and flame photometer method (NY/T 87-1988). The alkaline nitrogen content of PM was measured using the alkaline hydrolysis diffusion method [32]. The available potassium content of PM was measured by the ammonium acetate extraction and flame photometer method [33].

### 2.3. Analysis of Non-Volatile Compounds

For non-volatile metabolite profiling, an aliquot of 100 μL of each baijiu sample was mixed with an equal volume of 80% aqueous methanol, vortexed thoroughly, and incubated on ice for 5 min. The mixture was then centrifuged at 15,000× *g* and 4 °C for 20 min, and the supernatant was collected. To minimize methanol-induced matrix effects, the supernatant was diluted with LC-MS-grade water and subjected to a second centrifugation under identical conditions (4 °C, 15,000× *g*, 20 min). The final supernatant was subsequently collected for liquid chromatography-tandem mass spectrometry (LC-MS/MS) analysis. Each sample group includes three biological replicates.

LC-MS/MS analysis was performed using a Q Exactive™ HF-X mass spectrometer (Thermo Fisher Scientific, Braunschweig, Germany) coupled with a Vanquish UHPLC system (Thermo Fisher Scientific, Braunschweig, Germany) operating in both negative and positive ion modes. Chromatographic separation was achieved on a Hypersil Gold C18 column (100 × 2.1 mm, 1.9 μm) at 40 °C, with a flow rate of 0.2 mL/min. The mobile phase consisted of 0.1% formic acid in water (A) and methanol (B). Gradient elution was performed as follows: The mobile phase was initially composed of 98% A (2% B), held for 1.5 min, then linearly changed to 15% A (85% B) at 3 min and further to 0% A (100% B) at 10 min. The composition was then rapidly returned to 98% A (2% B) at 10.1 min and maintained until 12 min for column re-equilibration. The scanning mode was electrospray ionization (ESI) mode, with a spray voltage of 3.5 kV, a sheath gas flow rate of 35 psi, an auxiliary gas flow rate of 10 L/min, an ion transfer tube temperature of 320 °C, and an auxiliary gas heater temperature of 350 °C. Both the sheath gas and auxiliary gas used were nitrogen. To monitor instrument performance and analyze reproducibility, three quality control (QC) samples were inserted after every 20 test samples for continuous monitoring.

Raw mass spectrometry data were converted to mzXML format using ProteoWizard (v3.0) software, followed by peak picking, extraction, alignment, and quantitative integration using R and the XCMS package. Nonlinear retention time correction was performed across samples by matching retention times and mass-to-charge ratios (*m*/*z*). Prior to metabolite identification, a series of rigorous data filtering steps were implemented to eliminate systematic errors. First, ion features primarily originating from background contamination were removed by comparing peak areas in biological samples with those in blank samples. Subsequently, features with a relative standard deviation (RSD), i.e., a coefficient of variation greater than 30% in quality control (QC) samples, were excluded to remove unstable features affected by the high matrix effects of ethanol and instrumental fluctuations. In addition, internal standard peaks and all known false-positive peaks (including noise peaks, column bleed peaks, and derivatization reagent peaks) were further removed. The remaining valid features were deduplicated and merged. For the remaining features, ions with missing values exceeding 50% across all samples were filtered out, and the remaining missing values were imputed using the K-nearest neighbor (KNN) algorithm. Finally, the filtered peak area matrix was normalized using sum normalization to correct for differences in injection volume.

Metabolite identification was carried out using a comprehensive strategy that combined MS/MS spectral matching with accurate mass verification. First, MS1 features containing adduct ion information were matched against an in-house high-resolution MS/MS spectral database (NovoMetDB) with a stringent mass error threshold of ≤10 ppm. Subsequently, the putative identifications were further confirmed by thoroughly comparing the acquired MS/MS fragmentation spectra with those in the database and those reported in the literature. This ensured that the mass errors for all identified metabolites were strictly controlled within ±10 ppm. All annotations were manually checked and curated. According to the confidence level criteria of the Metabolomics Standards Initiative (MSI), only metabolites identified at level 2 or above were retained for subsequent statistical analysis.

The robustness and reliability of the metabolomics data were further validated through the following complementary approaches. First, the orthogonal partial least squares discriminant analysis (OPLS-DA) model exhibited a favorable goodness-of-fit and predictive ability, with R^2^Y values approaching 1.0 and Q^2^Y values exceeding 0.95. 200 permutation tests confirmed the absence of overfitting, as indicated by a negative Q^2^Y intercept. Second, the biological interpretability demonstrated by the key differential metabolites and their enriched pathways (e.g., unsaturated fatty acid biosynthesis, tryptophan metabolism) was consistent with the known metabolic characteristics of the SFB fermentation ecosystem, further supporting the biological validity of the dataset.

### 2.4. DNA Extraction, PCR Amplification, and Sequencing

5 g of PM sample was taken from each sample for DNA extraction and 16S rRNA sequencing. The extracted DNA was diluted with sterile water to a concentration of 1 ng/µL. After dilution, it was used to amplify the 16S rRNA region of the samples. The 16S V4 region primers (515F: GTGCCAGCMGCCGCGGTAA, 806R: GGACTACHVGGGTWTCTAAT) were used. All PCR mixtures contained 15 µL of Phusion High-Fidelity PCR Master Mix, 0.2 µM primers, and 10 ng of genomic DNA template. Initial denaturation was performed at 98 °C for 1 min, followed by 30 cycles of 98 °C (10 s), 50 °C (30 s), and 72 °C (30 s), with a final extension at 72 °C for 5 min. PCR products were purified using magnetic beads, pooled in equal amounts based on PCR product concentration, thoroughly mixed, and then detected to recover the target bands. The amplified PCR products were subjected to PCR product detection. Raw reads were preprocessed and analyzed using QIIME2. Denoising was performed using DADA2 in QIIME2 to obtain the final ASVs [34,35]. The Silva138.1 database in QIIME2 was used for analysis to generate corresponding species classifications. This study used primers 515F/806R to amplify the V4 hypervariable region of the 16S rRNA gene in bacteria and archaea. During subsequent bioinformatics processing, all archaeal and bacterial ASVs were analyzed together in the same workflow.

### 2.5. Statistical Analysis

In this study, alpha-diversity and beta-diversity analyses were performed using QIIME 2 (v 2021.8) and the R package VEGAN. To ensure data quality, Good’s coverage > 0.999 was adopted as the criterion for sufficient sequencing depth, and samples below this threshold were excluded from further analysis. Principal coordinate analysis (PCoA) and non-metric multidimensional scaling (NMDS) were conducted using R (v 4.0.3) with the ade4 and ggplot2 packages, respectively. Linear discriminant analysis effect size (LEfSe) analysis (v 1.1) was used to identify characteristic microbial indicator taxa in PM. Pairwise comparisons were performed among the four groups, and a linear discriminant analysis (LDA) score threshold of 4.0 was applied for feature selection. Subsequently, functional gene prediction for bacterial and archaeal communities was carried out using PICRUSt2 (v 2.3.0). Finally, canonical correspondence analysis (CCA) was applied to elucidate the major environmental factors shaping the community structures of bacteria and archaea.

All experiments included three replicates per treatment group. Prior to analysis, the Shapiro–Wilk test was used for normality testing and Levene’s test for homogeneity of variance, followed by a one-way analysis of variance (ANOVA) and Tukey’s HSD test (*p* < 0.05). For CCA and Spearman correlation analysis, all correlations were subjected to multiple comparison correction using the false discovery rate (FDR), with statistical significance determined at corrected *p* < 0.05. Data visualization and figure preparation were performed using Origin 2024.

All experiments included three replicates per treatment group. Prior to analysis, normality was assessed using the Shapiro–Wilk test and homogeneity of variances was tested using Levene’s test, followed by a one-way analysis of variance (ANOVA) and Tukey’s HSD test (*p* < 0.05). For CCA and Spearman correlation analyses, multiple comparisons were corrected using the FDR (False Discovery Rate), and associations were considered significant at *p* < 0.05 after correction. Data visualization and graph creation were performed using Origin 2024, Microsoft Excel 2024, and R v4.1.2.

## 3. Results

### 3.1. High-Throughput Sequencing Analysis

**Figure 1:**
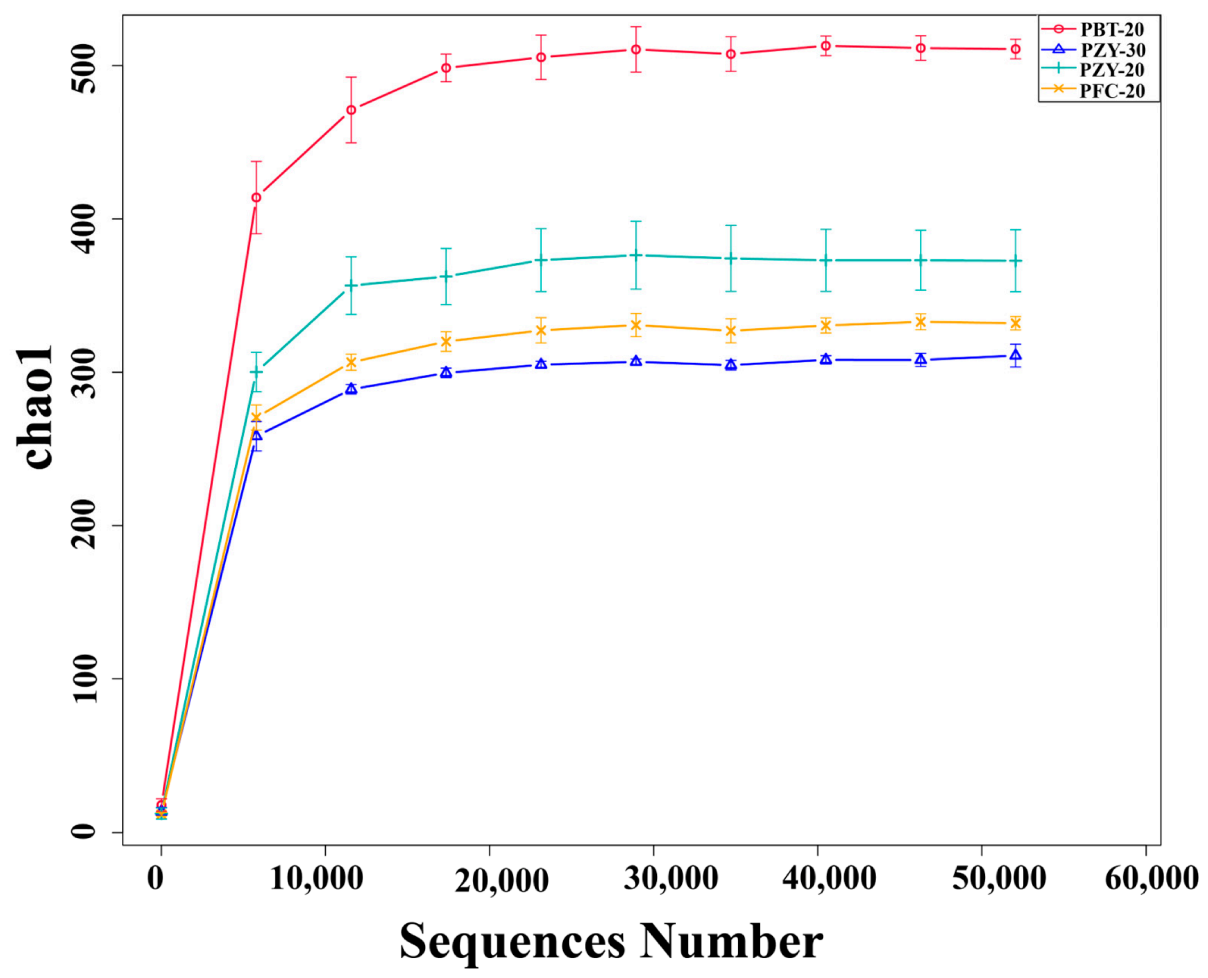
PBT-20 is the 20-year PM of Bitanchun Distillery, PZY-20 is the 20-year PM of Zhuyechun Distillery, PZY-30 is the 30-year PM of Zhuyechun Distillery, and PFC-20 is the 20-year PM of Fengchun Distillery.

As shown in Figure 1, the rarefaction curves of the four sets of different ASV samples indicate that the number of species increases with the number of sequencing reads. When the number of reads reaches 20,000, the sample numbers tend to stabilize. After DADA2 quality control, the 12 samples obtained an average of 104,386 high-quality sequences (with an average sequence length of 253.15 nt and an average Q30 of 97.41%), resulting in an average of 26,032 ASVs. This indicates that the samples are approaching saturation. The sequencing reads basically reflect the bacterial and archaeal community structure of PM and can be used to infer bacterial and archaeal diversity. In this study, the Good’s coverage of all samples was over 0.999, fully demonstrating that the sequencing depth is well above the threshold and can reliably support the subsequent diversity analysis.

### 3.2. Physicochemical Analysis of PM

**Figure 2:**
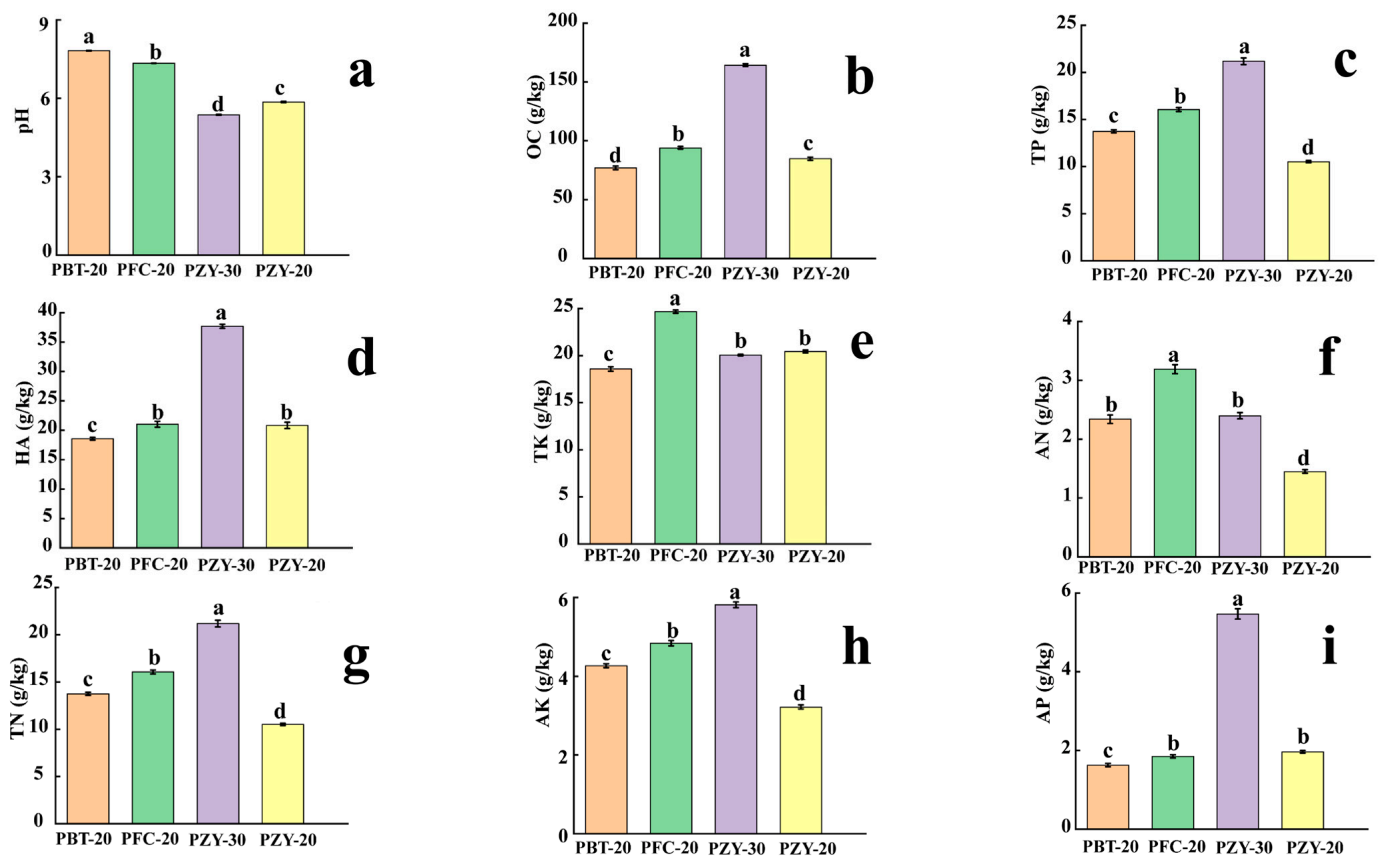
Physicochemical indicators in PM samples ((**a**) (pH); (**b**) (OC); (**c**) (TP); (**d**) (HA); (**e**) (TK); (**f**) (AN); (**g**) (TN)); (**h**) (AK); (**i**) (AP). Different letters indicate significant differences between samples (*p* < 0.05). PBT-20 is the 20-year PM of Bitanchun Distillery, PZY-20 is the 20-year PM of Zhuyechun Distillery, PZY-30 is the 30-year PM of Zhuyechun Distillery, and PFC-20 is the 20-year PM of Fengchun Distillery.

This study analyzed the contents of pH value (pH), organic carbon (OC), total phosphorus (TP), humic acid (HA), total potassium (TK), alkali-hydrolyzable nitrogen (AN), total nitrogen (TN), available potassium (AK), and available phosphorus (AP) in different PM samples, as shown in Figure 2. The pH content of PBT-20 was significantly higher than that of PFC-20 (*p* < 0.05), and the pH value of PFC-20 was close to neutral.

The contents of OC, TP, HA, TN, AN, AK, AP, and TK in PFC-20 were significantly higher than those in PBT-20 (*p* < 0.05). The pH content of PZY-20 was significantly higher than that of PZY-30 (*p* < 0.05), while the contents of OC, TP, HA, TN, AN, AK, AP, and other aspects in PZY-30 were significantly higher than those in PZY-20 (*p* < 0.05).

### 3.3. Diversity Analysis and Functional Prediction of Bacteria and Archaea

**Figure 3:**
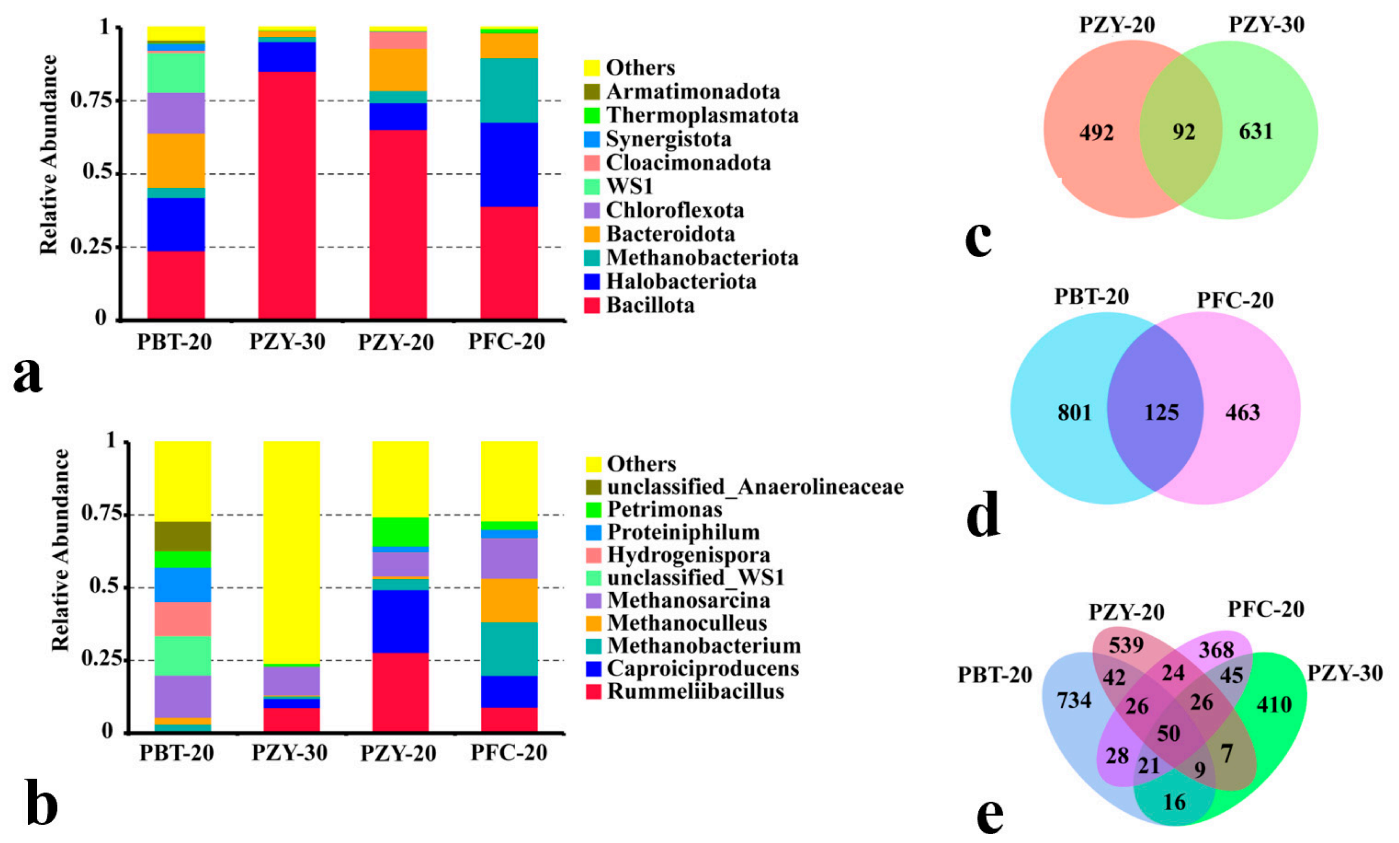
The top 10 most abundant phyla (**a**), genera (**b**), Venn diagrams (**c**,**d**), and petal diagrams (**e**) of different PMs. PBT-20 is the 20-year PM of Bitanchun Distillery, PZY-20 is the 20-year PM of Zhuyechun Distillery, PZY-30 is the 30-year PM of Zhuyechun Distillery, and PFC-20 is the 20-year PM of Fengchun Distillery.

The top 10 phyla and genera by relative abundance for different samples are shown in Figure 3a,b respectively. Different patterns emerged at the phylum level: a total of 35 bacterial and archaeal phyla were identified across all samples (Figure 3a). The most abundant phylum in all sample groups was *Bacillota*. As time progressed, the relative abundance of *Bacillota* in the PZY-30 sample increased further; the relative abundance of *Bacillota* was 65.31% in the PZY-20 sample and reached 85.24% in the PZY-30 sample. The relative abundance of *Bacillota* in the PBT-20 and PFC-20 samples was 23.98% and 39.15%, respectively. This was followed by a relative abundance of 28.65% for *Halobacteriota* in the PFC-20 sample and 18.47% for *Bacteroidota* in the PBT-20 sample.

At the genus level, a total of 341 bacterial and archaeal genera were identified across all samples (Figure 3b). The PM samples were enriched with four bacterial genera: *Rummeliibacillus*, *Caproiciproducens*, *Methanobacterium*, and *Methanosarcina*. Compared to PZY-30, the relative abundances of *Rummeliibacillus* and *Caproiciproducens* were significantly higher in PZY-20 (*p* < 0.05). *Methanosarcina* had the highest abundance in the PBT-20 sample, reaching 14.47%, while *Methanobacterium* was the most abundant in the PFC-20 sample, followed by *Caproiciproducens*.

The analysis of the study revealed the specific and shared ASVs among the four different distillery PM samples, as shown in Figure 3. PZY-20, PZY-30, PBT-20, and PFC-20 had 492, 631, 801, and 463 unique ASVs, respectively. PZY-20 and PZY-30 shared 92 ASVs (Figure 3c), PBT-20 and PFC-20 shared 125 ASVs (Figure 3d), and all samples shared 50 ASVs (Figure 3e).

**Figure 4:**
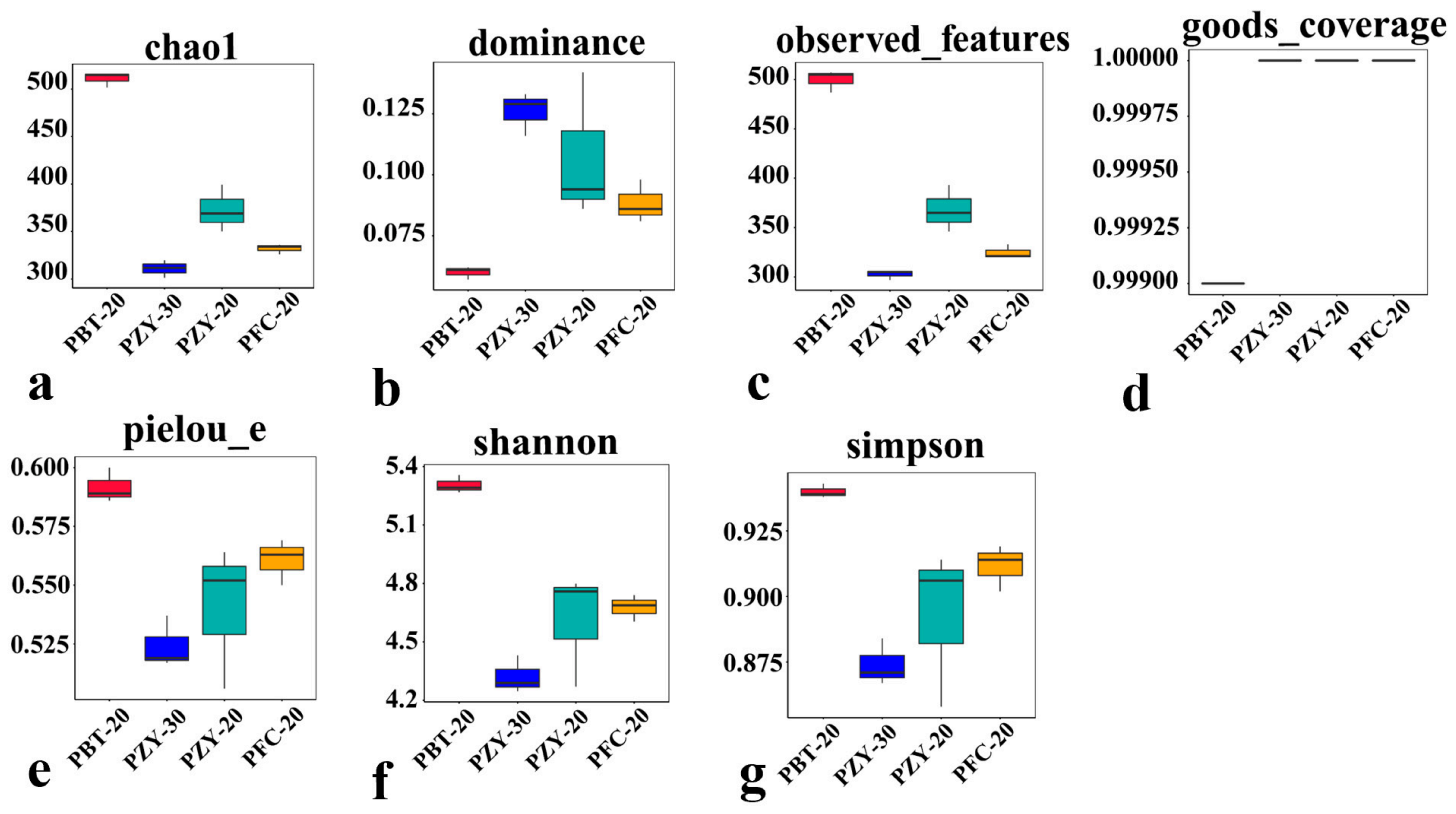
Box plots of alpha diversity indices for different PMs. chao1 (**a**), dominance (**b**), observed_features (**c**), goods_coverage (**d**), pielou_e (**e**), shannon (**f**), simpson (**g**). PBT-20 is the 20-year PM of Bitanchun Distillery, PZY-20 is the 20-year PM of Zhuyechun Distillery, PZY-30 is the 30-year PM of Zhuyechun Distillery, and PFC-20 is the 20-year PM of Fengchun Distillery.

Alpha diversity was assessed using the Chao1, dominance, observed_features, goods_coverage, pielou_e, shannon, simpson, and other indices to comprehensively evaluate the richness, evenness, and diversity of bacterial and archaeal communities in different PM samples (Figure 4). The Chao1, observed_features, and pielou_e values of PBT-20 were significantly higher than those of PFC-20, while the same three indices of PZY-30 were lower than those of PZY-20. In terms of diversity, PBT-20 had the highest Shannon and Simpson indices, while PZY-30 showed the lowest Shannon index and the highest Simpson dominance index. This indicates that a few dominant species dominated this sample, resulting in lower community evenness. Additionally, the Goods_coverage for all samples was above 0.999, showing that the sequencing depth was sufficient to accurately reflect the diversity and richness of bacteria and archaea in the samples.

**Figure 5:**
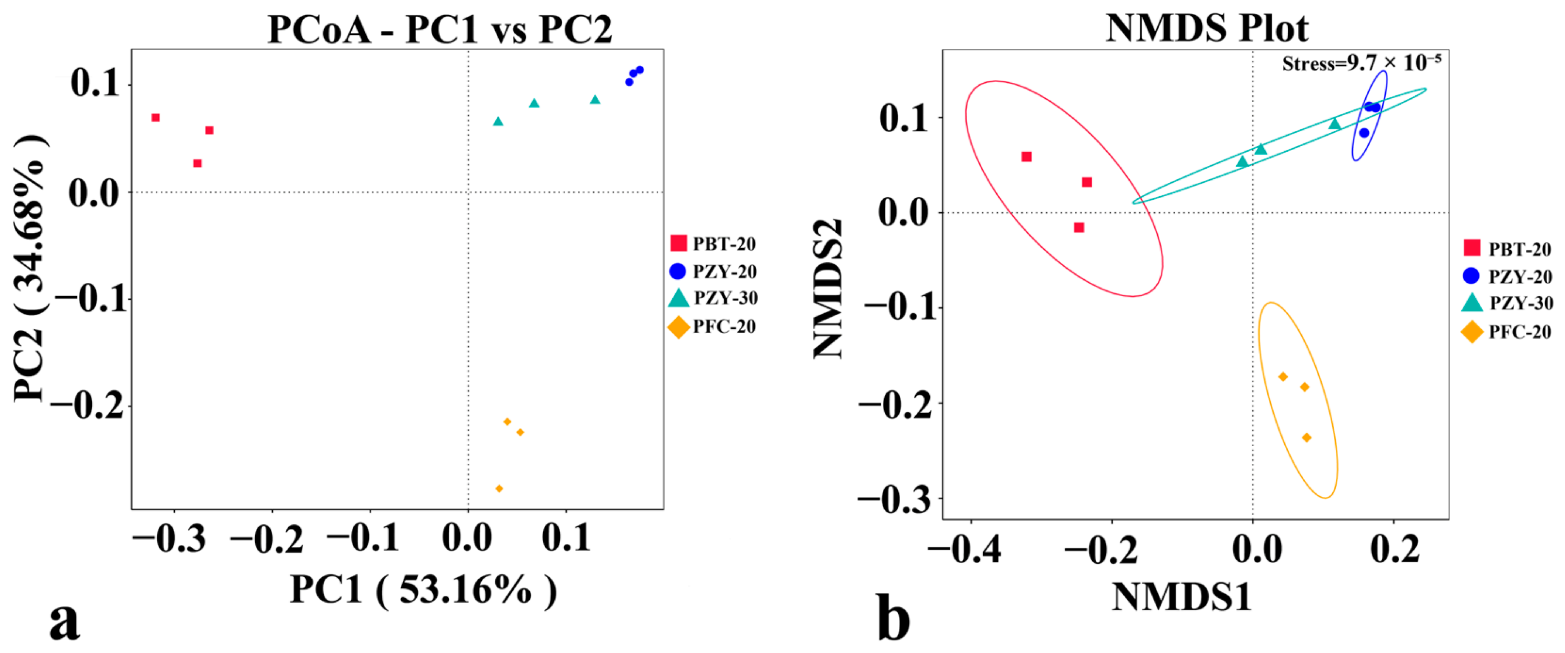
Shows the principal coordinate analysis (PCoA) of bacterial and archaeal communities based on the Weighted Unifrac distance matrix (**a**) and Unweighted Unifrac (**b**). PBT-20 is the 20-year PM of Bitanchun Distillery, PZY-20 is the 20-year PM of Zhuyechun Distillery, PZY-30 is the 30-year PM of Zhuyechun Distillery, and PFC-20 is the 20-year PM of Fengchun Distillery.

The PCoA analysis based on weighted UniFrac distance (Figure 5a) shows that samples from PZY-20 and PZY-30 cluster closely together, while PBT-20 and PFC-20 are clearly separated. NMDS analysis using unweighted UniFrac distance (Figure 5b) confirms this, suggesting that the microbial communities from the same distillery are similar, whereas those from different distilleries differ quite a bit.

**Figure 6:**
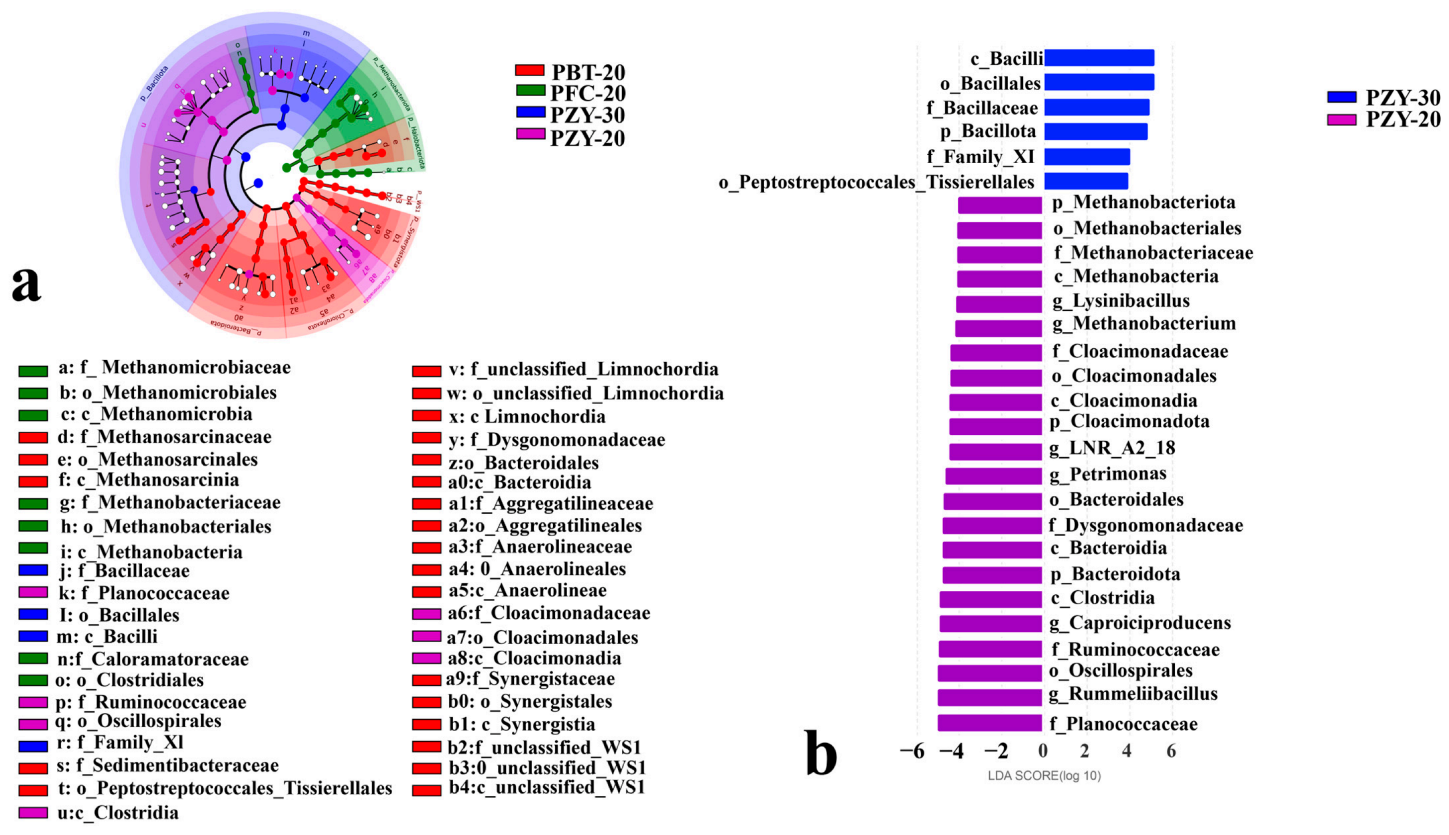
Evolutionary tree of microbial communities in PM samples from different years (**a**); LDA score distribution chart (**b**). PBT-20 is a 20-year-old PM from Bitanchun Distillery, PZY-20 is a 20-year-old PM from Zhuyechun Distillery, PZY-30 is a 30-year-old PM from Zhuyechun Distillery, and PFC-20 is a 20-year-old PM from Fengchun Distillery.

A comparison analysis was done on bacteria and archaea with LEfSe > 4, as shown in Figure 6. PBT-20 had higher relative abundances of *unclassified_WS1*; PFC-20 had higher abundances of *Methanobacteriales*, *Methanobacteriaceae*. PZY-30 had more abundant *Bacilli* and *Bacillales*, while PZY-20 showed more prominent abundances of *Ruminococcaceae*, *Oscillospirales*, *Rummeliibacillus*, and *Planococcaceae*.

**Figure 7:**
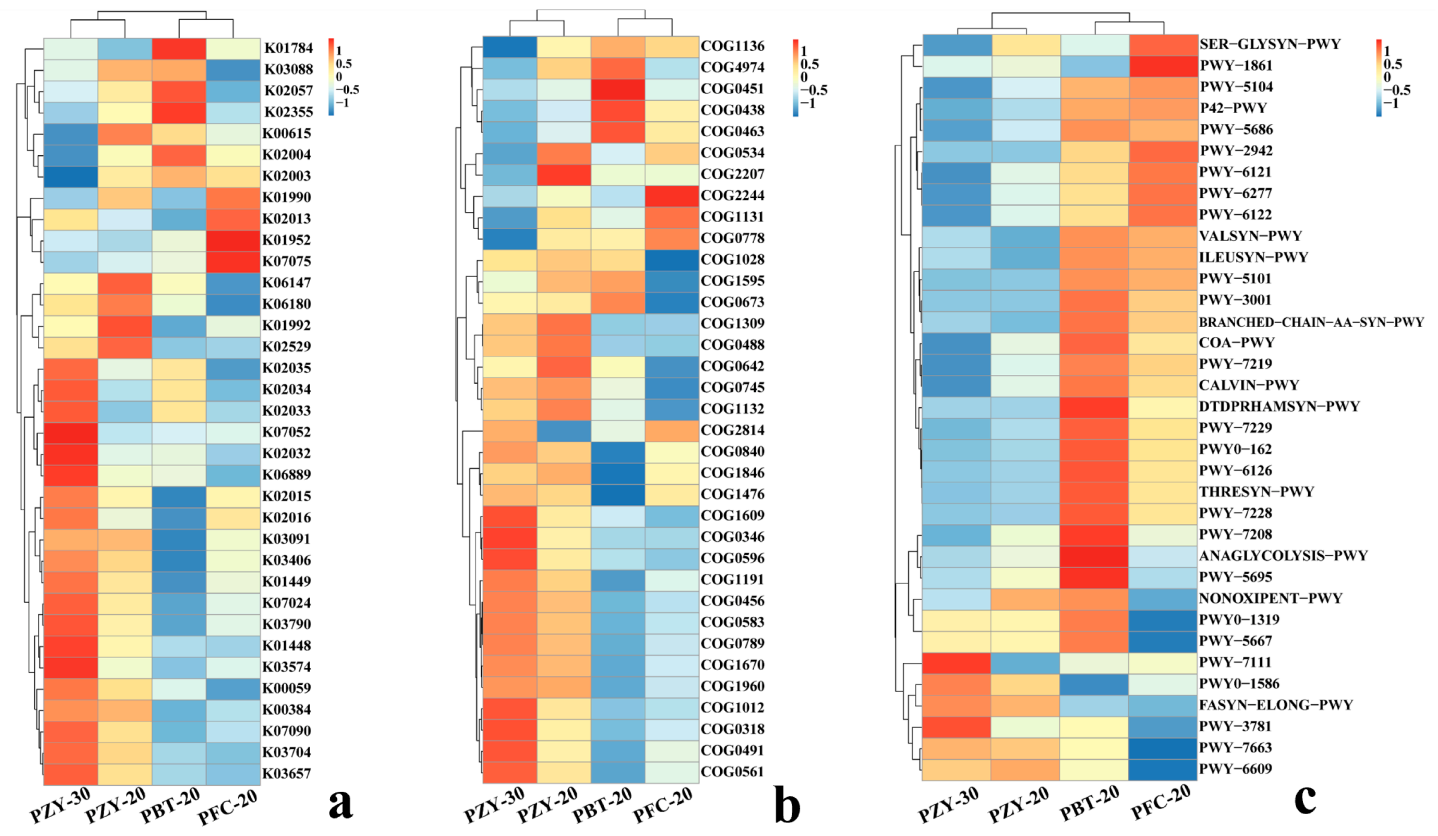
Functional predictions based on PICRUSt2 for KO (**a**), COG (**b**), and MetaCyc (**c**). Different colors represent varying relative abundances, with blue to red indicating an increase in relative abundance. PBT-20 is the 20-year PM of Bitanchun Distillery, PZY-20 is the 20-year PM of Zhuyechun Distillery, PZY-30 is the 30-year PM of Zhuyechun Distillery, and PFC-20 is the 20-year PM of Fengchun Distillery.

We predicted the bacterial and archaeal community functions of PM based on the PICRUSt2 database. In Figure 7a of the KEGG database, compared to the PFC-20 group, the PBT-20 group showed upregulation of the UDP-glucose 4-epimerase (K01784) function, while the RNA polymerase sigma-E/F/G factor (K03091) and methyl-accepting chemotaxis protein (K03406) functions were downregulated. In contrast, the PFC-20 group exhibited upregulation of the phosphoribosylformylglycinamidine synthase (K01952) function, while the RNA polymerase sigma-70 factor, ECF subfamily (K03088) function was downregulated. Compared to the PZY-20 group, the PZY-30 group showed upregulation of the CAAX protease family protein (K07052) function, while the putative ABC transport system ATP-binding protein (K02003) function was downregulated. The PZY-20 group displayed upregulation of the ABC-2 type transport system permease protein (K01992) function, while the peptide/nickel transport system permease protein (K02033) function was downregulated.

In Figure 7b of the COG database, compared to the PFC-20 group, the PBT-20 group showed an upregulation in the function of nucleoside-diphosphate-sugar epimerase (COG0451), while the functions of DNA-binding transcriptional regulator, MarR family (COG1846), and DNA-binding transcriptional regulator, XRE-family HTH domain (COG1476) were downregulated. In the PFC-20 group, the function of the membrane protein involved in the export of O-antigen and teichoic acid (COG2244) was upregulated, while the function of NAD(P)-dependent dehydrogenase of the short-chain alcohol dehydrogenase family (COG1028) was downregulated. Compared to the PZY-20 group, the PZY-30 group showed upregulation of DNA-binding transcriptional regulator, LacI/PurR family (COG1609), glyoxalase, catechol 2,3-dioxygenase or related enzyme, vicinal oxygen chelate (VOC) family (COG0346), and 2-succinyl-6-hydroxy-2,4-cyclohexadiene-1-carboxylate synthase MenH/undecaprenyl monophosphate-sugar esterase UshA/YqjL (COG0596) functions, while downregulation of ABC-type lipoprotein targeting system ATPase component LolD (COG1136) and Nitroreductase (COG0778) functions was observed. In the PZY-20 group, the AraC-type DNA-binding domain and AraC-containing proteins (COG2207) function were upregulated, while the predicted arabinose efflux permease AraJ, MFS family (COG2814) function was downregulated.

In Figure 7c of the PWY database, compared to the PFC-20 group, the PBT-20 group showed potential upregulation in the functions of glycolysis III (ANAGLYCOLYSIS-PWY) and purine-nucleoside phosphorylase (PWY-5695), while there was a downward trend in the function of formaldehyde assimilation II (PWY-1861). Interestingly, the PFC-20 group exhibited a possible upregulation in the function of formaldehyde assimilation II (PWY-1861), while the functions of CDP-diacylglycerol biosynthesis II (PWY0-1319) and CDP-diacylglycerol biosynthesis I (PWY-5667) were downregulated. Compared to the PZY-20 group, the PZY-30 group showed upregulated function in pyruvate fermentation to isobutanol (PWY-711), while the superpathway of L-serine and glycine biosynthesis I (SER-GLYSYN-PWY) function was downregulated. The PZY-20 group and the PZY-30 group shared an upregulated common function of fatty acid elongation saturated (FASYN-ELONG-PW); however, compared to the PZY-30 group, the PZY-20 group exhibited a significant downregulation in the function of pyruvate fermentation to isobutanol (PWY-7111).

### 3.4. Non-Targeted Metabolomic Analysis of Baijiu

This study employed LC-MS-based untargeted metabolomics to identify metabolites in Baijiu, screening differential metabolites between different Baijiu types as shown in Supplementary Figure S1. Based on HMDB, 1206 non-volatile ion signals were identified, including 1001 metabolites in positive ion mode and 205 in negative ion mode. These encompassed lipids and lipid-like molecules (30.88%), organic heterocyclic compounds (18.57%), organic acids and derivatives (13.72%), benzenoids (11.30%), phenylpropanoids and polyketides (8.78%), among others.

### 3.5. OPLS-DA Analysis

**Figure 8:**
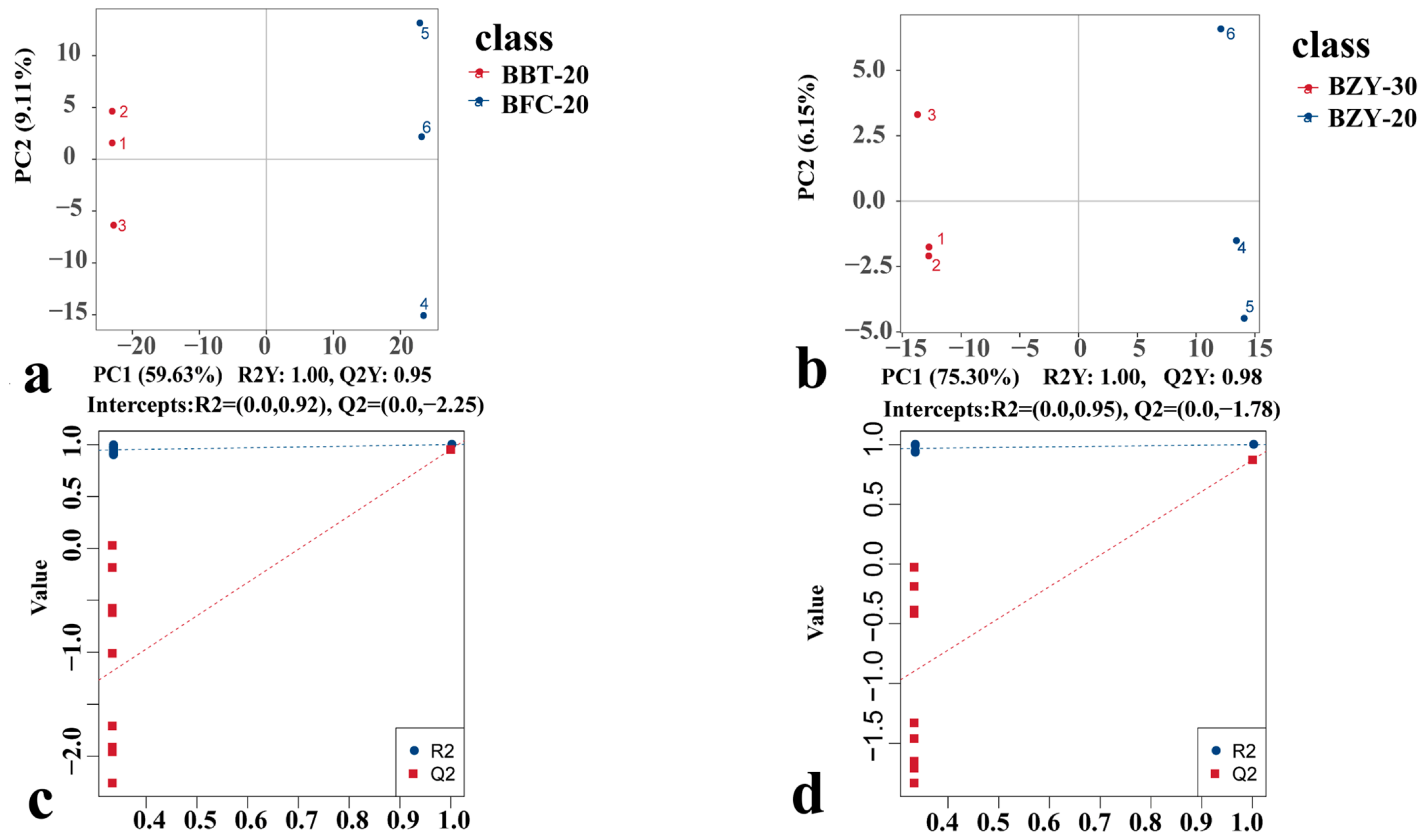
(**a**,**b**) are principal component analysis plots, while (**c**,**d**) are permutation test plots. BBT-20 is a 20-year-old baijiu from Bitanchun Distillery. BZY-20 is a 20-year-old baijiu from Zhuyechun Distillery. BZY-30 is a 30-year-old baijiu from Zhuyechun Distillery and BFC-20 is a 20-year-old baijiu from Fengchun Distillery.

OPLS-DA is a supervised learning method used for classification and discriminant analysis (see Figure 8). In this study, partial least squares regression was used to create a predictive model between metabolite expression and sample categories [36]. Through 200 permutation test iterations and cross-validated permutation testing, the model for the PBT-20 group and PFC-20 group has an R2Y of 1.00 and a Q2Y of 0.95. The y intercept of R2 was 0.92, and the y intercept of Q2 was −2.25. The model for the PZY-30 group and PZY-20 group had an R2Y of 1.00 and a Q2Y of 0.98. The y intercept of R2 was 0.95, and the y intercept of Q2 was −1.78. This indicates significant intergroup differences between the two sample groups above, with R2Y and Q2Y demonstrating good fit and predictive ability between groups. The OPLS-DA model can clearly distinguish the two sample groups. Furthermore, the permutation test results show that neither model exhibits overfitting, confirming the reliability of intergroup differences and the statistical significance of the results.

### 3.6. Comparative Analysis of Differential Metabolites in Baijiu

**Figure 9:**
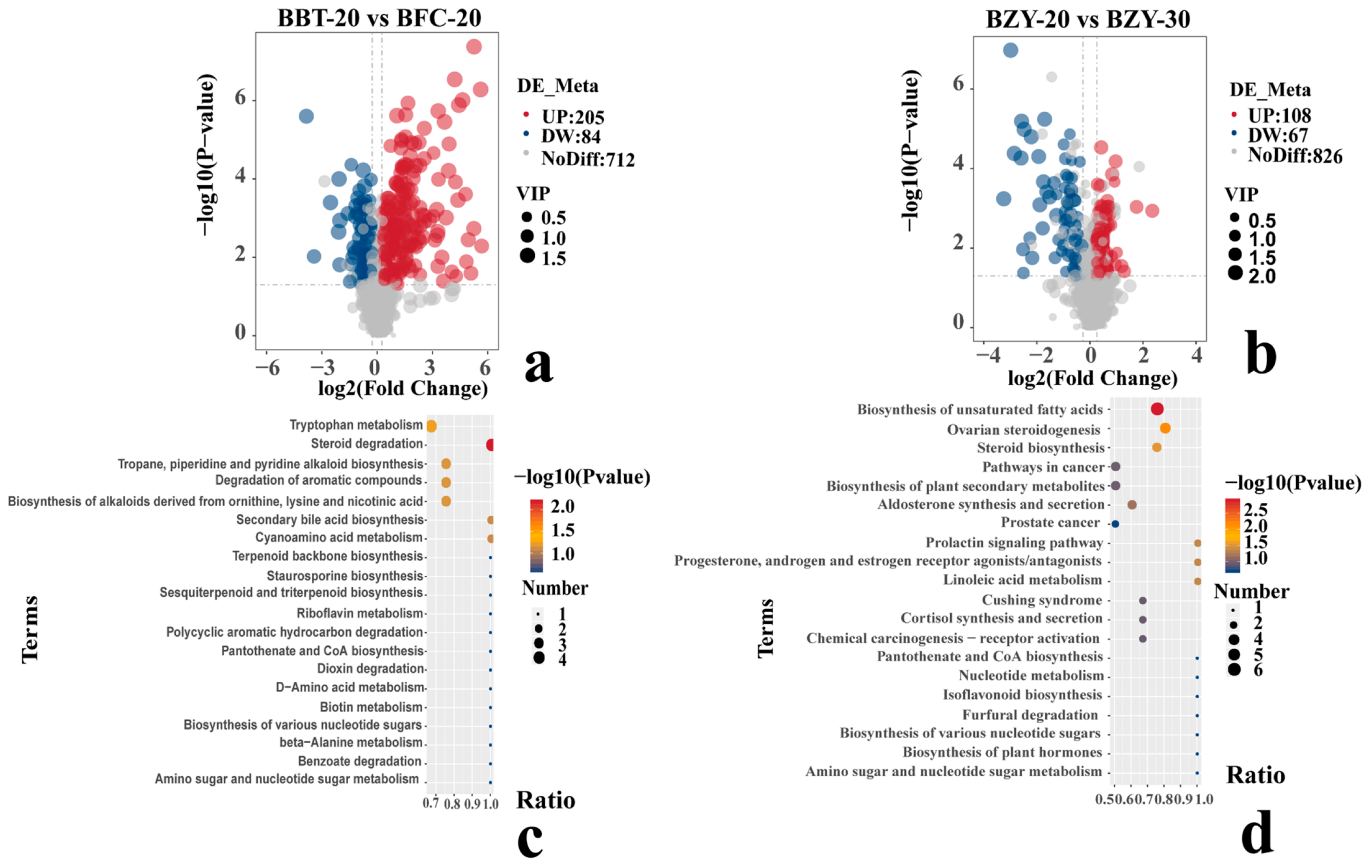
Volcano plots showing the different metabolites between baijiu samples (**a**,**b**), and bubble plots of the enriched differential metabolites (**c**,**d**). BBT-20 is a 20-year-old baijiu from Bitanchun Distillery. BZY-20 is a 20-year-old baijiu from Zhuyechun Distillery. BZY-30 is a 30-year-old baijiu from Zhuyechun Distillery and BFC-20 is a 20-year-old baijiu from Fengchun Distillery.

To further analyze the differential metabolites among the four groups of Baijiu, the following volcano plot was presented (Figure 9) [37]. The differential metabolites from different distilleries were compared: BBT-20 vs. BFC-20, and BZY-20 vs. BZY-30. Metabolites with significant changes were highlighted (VIP > 1, FC > 1.2, *p* < 0.05). In Figure 9a, there were 289 positive ion differential metabolites between BBT-20 and BFC-20 samples, with 205 metabolites upregulated and 84 downregulated. In Figure 9b, 175 positive ion mode metabolites were significant in BZY-20 vs. BZY-30 samples, with 108 metabolites upregulated and 67 downregulated.

To analyze the potential enrichment of differential metabolic pathways, the KEGG database was selected. This database provides *p*-values for the identification of enriched pathways. The *p*-value is negatively correlated with enrichment, meaning that the smaller the *p*-value, the more significant the enrichment. The size of the bubbles represents the number of differential metabolites in the corresponding pathway. The larger the bubble, the more differential metabolites are present in that pathway. In Figure 9c, tryptophan metabolism was the most significantly enriched. SFB samples showed enrichment in tropane, piperidine, and pyridine alkaloid biosynthesis, degradation of aromatic compounds, terpenoid backbone biosynthesis, and steroid degradation. The BBT-20 and BFC-20 group samples are pivotal in mediating alkaloid synthesis, steroid degradation. Among these, steroid degradation had the highest number of differential metabolites. The biosynthesis of alkaloids derived from ornithine, lysine and nicotinic acid is completed in the early stages by PM microorganisms during solid-state fermentation. Its precursor, putrescine, regulates microbial osmotic pressure and stabilizes the microbial communities in new and old fermentation pits [38]. Additionally, microorganisms such as *Saccharomyces cerevisiae* and Actinobacteria synergistically regulate the terpenoid backbone biosynthesis pathway and play a critical role in the formation of Baijiu flavor [39,40]. In old PM, microbial communities such as Bacillus, *Clostridium*, methanogens, and sulfur-reducing bacteria can degrade steroids into small-molecule acids and aromatic compounds [41].

Figure 9d shows that the biosynthesis of unsaturated fatty acids contains the highest number of differential metabolites. The biosynthesis of unsaturated fatty acids is a critical pathway for the maturation of PM in SFB and the formation of flavor compounds. It serves as a precursor for key ester aromas such as ethyl hexanoate and ethyl butyrate, and also influences the softness and richness of the liquor body [42]. The enrichment of plant secondary metabolite pathways indicates that sorghum and wheat raw materials from earlier stages are utilized and transformed by microorganisms during fermentation, leading to the formation of functional compounds [43].

### 3.7. Correlation Analysis

**Figure 10:**
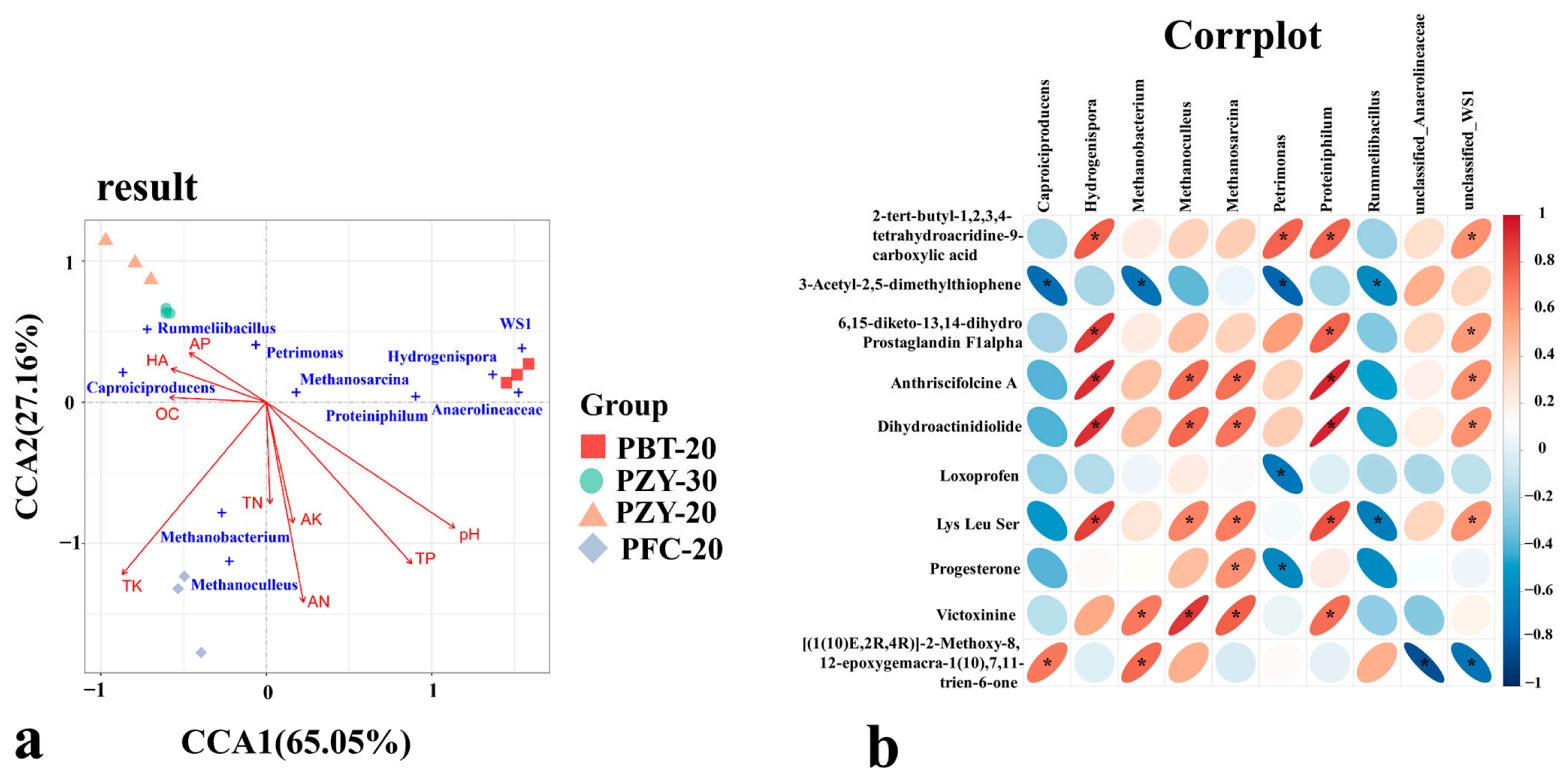
(**a**) Shows the correlation between bacteria, archaea, and the physicochemical factors of the PM. Red arrows indicate different influencing factors. (**b**) shows a heatmap of the correlation between bacteria and key differential metabolites. Asterisks indicate significance (*p* < 0.05). PBT-20 is the 20-year PM of Bitanchun Distillery, PZY-20 is the 20-year PM of Zhuyechun Distillery, PZY-30 is the 30-year PM of Zhuyechun Distillery, and PFC-20 is the 20-year PM of Fengchun Distillery.

Using canonical correspondence analysis, we selected bacteria and archaea at the genus level to speculate on their correlation with environmental factors. As shown in Figure 10a, CCA1 and CCA2 explain 65.05% and 27.16% of the total variance, respectively. TN, AN, AK, and TK may be positively correlated with *Methanobacterium* and *Methanoculleus* in PFC-20. HA, AP, and OC may be positively correlated with *Caproiciproducens* and *Rummeliibacillus* in PZY-20 and PZY-30. TP, AP, HA, and AN may be negatively correlated with *Hydrogenispora*, *Anaerolineaceae*, and *Proteiniphilum* in PBT-20.

A Spearman correlation analysis was conducted using bacteria and archaea at the genus level to infer correlations with metabolites. As shown in Figure 10b, 3-Acetyl-2,5-dimethylthiophene may show a negative correlation with *Caproiciproducens*, *Methanobacterium*, *Rummelibacillus*, and *Petrimonas*. Dihydroactinidiolide, Lys Leu Ser, and *Hydrogenispora*, *Methanoculleus*, *Methanosarcina*, *Proteiniphilum*, and *unclassified_WS1* may show a positive correlation. [(1(10)E,2R,4R)1-2-Methoxy-8,12-epoxygemacra-1(10),7,11-trien-6-one may show a positive correlation with *Methanobacterium* and *Caproiciproducens*.

## 4. Discussion

### 4.1. Physical and Chemical Factors Influence the Succession of Bacterial and Archaeal Communities in PM

The pit is enriched with abundant bacteria and archaea, and changes in physicochemical indicators are crucial for their development and succession [44]. Variations in the pH of PM can influence the activities of bacteria and archaea [45]. As shown in Figure 2a, the near-neutral pH of PFC-20 was more conducive to Baijiu production, favoring microbial proliferation, promoting ethanol production, and enhancing the synthesis of flavor compounds, which is consistent with previous findings [46]. Compared with the PFC-20 group, the weakly alkaline pH of the PBT-20 group may favor the growth of acid-sensitive bacteria, such as *Clostridium* and *Sedimentibacter* [47,48]. In contrast, the pH of the PZY-30 group was significantly lower than that of the PZY-20 group, which may be associated with the degradation of PM and a potential decline in its quality [49]. Among these indicators, HA, as an important marker of PM maturity, can provide trace elements and nutrients for the PM microbiota [50]. The HA content in the PZY-30 group was significantly higher than that in the PZY-20 group (*p* < 0.05), which may be attributed to the prolonged domestication of the PM. Mature PM exhibits a stronger capacity for moisture retention and nutrient supply, effectively alleviating PM hardening. The accumulation of humic acid favors the growth and metabolism of functional bacteria, such as caproic acid-producing bacteria and butyric acid-producing bacteria, thereby improving the quality of SFB [51]. The OC, TP, TN, AP, AK, and AN levels in the PZY-30 group showed consistent trends, indicating that the PZY-30 PM possessed sufficient organic matter and nutrients to effectively support microbial growth and metabolism.

### 4.2. Analysis of Bacterial and Archaeal Diversity and Functional Prediction

The microorganisms in PM of different pit ages have formed stable microbial communities through long-term selection with the surrounding environment. At the genus level, *Rummeliibacillus*, *Caproiciproducens*, *Methanobacterium*, and *Methanosarcina* are the dominant genera in PM samples. These are also functional bacteria and archaea in strong-flavor PM, influencing the quality of Baijiu [52,53]. As the PM age increases, the relative abundance of *Caproiciproducens* and *Methanosarcina* rises, suggesting that these bacteria and archaea can independently adapt to the environment of the PM-30 sample group over the long term, which is consistent with previous results [54]. *Rummeliibacillus*, in a synergistic fermentation with multiple microorganisms, is applied in SFB production, leading to a significant increase in ethyl hexanoate content compared to pre-inoculation levels [55]. *Caproiciproducens* can utilize lactic acid to assist in the production of hexanoic acid. This process is influenced by the depth of PM and serves as a key precursor for ethyl hexanoate, the typical flavor compound found in SFB [29].

Compared to the PFC-20 group, the PBT-20 group exhibited higher Chao1 and observed_features indices, which may be related to the carbon sources, nitrogen sources, and trace elements present, providing growth opportunities for various microorganisms, such as *Anaerolineaceae*. Compared to the PZY-20 group, the PZY-30 group showed lower abundance, possibly due to the microbial community having already transitioned to a stable and complex microbial system during the fermentation process, filtering out microbial communities with low stress resistance and accumulating symbiotic and mutualistic functional groups. Additionally, the dominance index indicates that a few functional species dominate the PZY-30 group. Notably, the species abundance in the PBT-20 group is the most uniform, with a balanced nutrient distribution among different microbial communities, effectively preventing the overexpansion of single species.

Compared with the PFC-20 group, the predicted function K03091 (RNA polymerase sigma-E/F/G factor) was downregulated in the PBT-20 group, which may be associated with the environmental sensitivity of *Hydrogenispora*, *Anaerolineaceae*, and *Proteiniphilum*. Under conditions of high nutrient availability and pronounced pH fluctuations, the RNA polymerase-mediated directional expression of genes related to extreme environmental adaptation may be reduced [56]. Compared with the PBT-20 group, the predicted functions PWY-1861 (formaldehyde assimilation II) and FASYN-ELONG-PWY (fatty acid elongation–saturated) were potentially upregulated in the PFC-20 group. These anaerobic methanogenic archaea, such as *Methanobacterium* and *Methanoculleus*, may promote the elongation of saturated fatty acids in the cell membrane, thereby maintaining methanogenic metabolic activity and enhancing their stability and enrichment under high AN, AK, and high pH conditions [57].

Compared with the PZY-20 group, the predicted function K07052 (CAAX protease family protein) was upregulated in the PZY-30 group. This gene may assist methanogenic archaea in energy metabolism transfer, ensuring the energy supply for methanogenic archaea [58]. Compared with the PZY-30 group, the predicted function COG2207 (AraC-type DNA-binding domain and AraC-containing proteins) was potentially upregulated in the PZY-20 group. This may promote the expression of stress protein genes, helping *Rummeliibacillus* and *Caproiciproducens* tolerate high concentrations of humic acid and adverse environmental conditions. This, in turn, maintains normal microbial metabolic activities [59].

This study is based on PICRUSt for microbial community functional annotation and pathway prediction, and the results reflect the potential functions of microbial genomes. Future studies can design auxiliary validations targeting key genes and metabolic pathways, providing more accurate evidence through molecular detection, strain cultivation, and other methods.

### 4.3. Differential Metabolites of SFB

Non-volatile compounds contribute significantly to taste perception. The non-volatile metabolites in Baijiu were predominantly lipids and lipid-like molecules (30.88%), as shown in Figure S1. Previous studies have demonstrated that during Baijiu fermentation, lipids in PM undergo hydrolysis and oxidative degradation, generating a large number of volatile flavor compounds (such as fatty acids and esters) and non-volatile flavor precursors, which represent one of the core sources contributing to the characteristic aroma formation of Baijiu [60]. The predominance of lipids and lipid-like molecules as the most abundant metabolites may be related to the starter culture (Qu) used in the fermentation process [61]. Representative compounds include free fatty acids (e.g., linoleic acid, oleic acid, palmitic acid), lysophospholipids, and fungal sterol derivatives (e.g., ergosterol). During Baijiu fermentation, lipids and their derivatives directly participate in the biosynthetic pathways of ester aroma compounds through hydrolysis, oxidation, and esterification reactions. Furthermore, lipid-soluble antioxidant components such as sterols and vitamin E can effectively scavenge free radicals within the system, delay the oxidative deterioration of aroma compounds, and reduce their volatilization rate, thereby exerting a positive regulatory effect on flavor fidelity and storage stability of aged Baijiu. As shown in Figure 9b, the BBT-20 vs. BFC-20 comparison revealed a significantly higher number of upregulated metabolites compared to downregulated metabolites, indicating that the BBT-20 group had a significant enrichment of upregulated differential metabolites (*p* < 0.05). In the comparison between BZY-20 and BZY-30, the number of upregulated metabolites was significantly higher than that of downregulated metabolites, indicating significant differences between the BZY-20 and BZY-30 groups (*p* < 0.05). As shown in Figure 9b (BBT-20 vs. BFC-20), the number of upregulated metabolites is significantly higher than that of downregulated metabolites, indicating that BBT-20 significantly enriches upregulated differential metabolites (*p* < 0.05). For BZY-20 vs. BZY-30, the number of upregulated metabolites is significantly higher than that of downregulated metabolites, indicating a significant difference between BZY-20 and BZY-30 (*p* < 0.05).

### 4.4. Effects of Bacterial and Archaeal Communities Driven by Pit Mud Physicochemical Factors on Baijiu Metabolites

Changes in the physicochemical indicators of PM can affect the bacterial and archaeal communities, and the differences in these communities may, in turn, influence the metabolites in SFB. These three factors interact with and constrain one another [62]. CCA results revealed that pH, TP, AN, and TK were the key environmental physicochemical factors driving the succession of bacterial and archaeal communities. In the PBT-20 samples, *Anaerolineaceae* and *Hydrogenispora* were potentially positively correlated with pH and TP. *Methanosarcina* may have been negatively correlated with AP, HA, and OC. Studies have shown that methanogenic archaea, such as *Methanosarcina* and *Methanobacterium*, form a syntrophic symbiotic system with bacteria that can effectively improve metabolic efficiency and construct a metabolic cross-feeding network [63,64]. The *Anaerolineaceae* family functions in carbohydrate and protein metabolism, and its metabolic products may include hydrogen [65]. In the PZY-20 and PZY-30 samples, *Rummeliibacillus* and *Caproiciproducens* were potentially positively correlated with OC, HA, and AP, while the microbial community in the PZY-30 samples may have been reduced, with a concentrated enrichment of microorganisms growing in high OC and high HA environments, forming a unique survival pattern.

The Spearman correlation analysis heatmap revealed that dihydroactinidiolide and Lys Leu Ser were potentially positively correlated with *Hydrogenispora*, *Methanoculleus*, *Methanosarcina*, *Proteiniphilum*, and *unclassified_WS1* in PM. Combined with the CCA results, these microbial groups were primarily enriched in PM environments with sufficient nitrogen and phosphorus nutrient supply, where pH, TN, AN, TP, and AK were the core environmental factors driving the above-mentioned archaea and bacteria. The synergistic interactions among these microbial groups formed a mature anaerobic syntrophic metabolic network. Specifically, *Hydrogenispora* is an obligately anaerobic, hydrogen-producing Clostridium that intracellularly accumulates various reducing substances such as NADH and H_2_, along with a diverse enzyme system. This enables the efficient decomposition of macromolecular organic matter and provides sufficient carbon skeletons and reducing power for the synthesis of terpene lactones [66]. *Proteiniphilum* possesses excellent proteolytic capacity, hydrolyzing macromolecular proteins and long-chain peptides to release free amino acids that subsequently condense to form small-molecule oligopeptides. The process ensures the accumulation of Lys Leu Ser [67]. *Methanoculleus* and *Methanosarcina*, as dominant methanogenic archaea, may maintain the stability of the metabolic environment by integrating fatty acid chain elongation, consuming H_2_ and organic acids within the system, alleviating anaerobic metabolic inhibition, and stabilizing the pH of the metabolic environment, thereby safeguarding the smooth operation of the metabolic pathways of upstream fermentative microorganisms [68].

3-Acetyl-2,5-dimethylthiophene may show a negative correlation with *Caproiciproducens*, *Rummeliibacillus*, *Methanobacterium*, and *Petrimonas*. 3-Acetyl-2,5-dimethylthiophene is a key trace sulfur-containing heterocyclic aroma compound in baijiu. The aforementioned bacterial communities may utilize substrates such as sulfur-containing amino acids and sugars within the system during their growth and metabolism, thereby consuming the precursors required for the synthesis of thiophene compounds. Furthermore, the hydrogenotrophic methanogenic archaeon *Methanobacterium* is involved in anaerobic carbon metabolism. This bacterium may exhibit a significant negative correlation with 3-acetyl-2,5-dimethylthiophene, possibly due to the consumption of H_2_ and NADPH during its metabolic processes [69].

The CCA results indicated that pH, TP, AN, and TK were key environmental factors associated with the succession of bacterial and archaeal communities. The interrelationships among different bacteria, archaea, and environmental factors reflect, to a certain extent, the shaping effect of environmental conditions on microbial community structure. The discussion is based on correlation analysis, and the underlying mechanisms await further experimental verification.

## 5. Conclusions

This study reveals the composition and functional characteristics of bacterial and archaeal communities in PMs under different distilleries and pit ages, and their impact on the differential metabolites of baijiu. The results showed that after multiple fermentation cycles, the bacterial diversity and physicochemical indices exhibited variations among different distilleries. The PM was significantly enriched with core functional bacteria such as *Rummeliibacillus*, *Caproiciproducens*, *Methanobacterium*, and *Methanosarcina* that synergistically interacted to drive the maturation of the PM. Functional prediction analysis revealed that the methanogens in PZY-30 upregulated the functional gene K07052, while the PM from PFC-20 activated the metabolic pathways PWY-1861 and FASYN-ELONG-PWY, maintaining their colonization stability and metabolic activity in the PM microenvironment. Furthermore, this study revealed the correlation between functional microbial communities and differential metabolites of Baijiu. The findings offer a theoretical reference for exploring the rules of Baijiu flavor formation and guiding the production of high-quality Baijiu.

## Data Availability

The datasets generated in this study, including raw 16S rRNA amplicon sequences, ASV tables processed using QIIME2, and raw LC-MS/MS files, have been deposited in the NCBI Sequence Read Archive (SRA) under accession number PRJNA1482013. The original contributions presented in this study are included in the article/Supplementary Material. Further inquiries can be directed to the corresponding authors.

## Supplementary Materials

The following supporting information can be downloaded at: https://www.mdpi.com/article/10.3390/foods15152657/s1. Supplementary Figure S1: Main classification of metabolites.
