# Supplementary material for: Variations in Bacterial and Archaeal Communities and Baijiu Metabolites by Distillery and Pit Age
Source: Foods. 2026 Jul 28;15(15):2657. doi: 10.3390/foods15152657 (PMC13464765; doi:10.3390/foods15152657)
Supplement: Supplementary file 1 [file foods-15-02657-s001.zip › foods-4434077-supplementary.pdf]

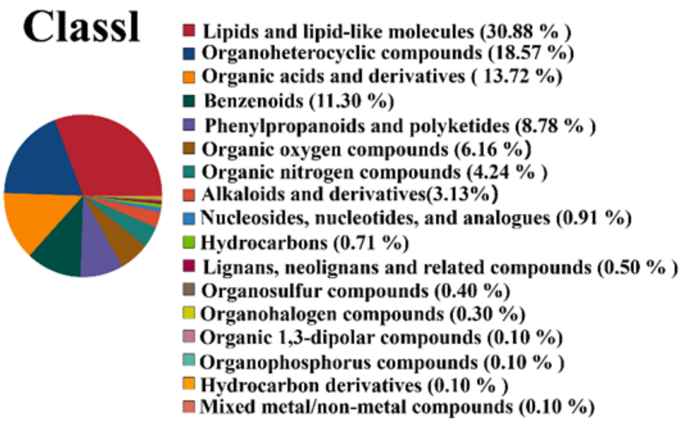

Figure S1. Main classification of metabolites.
